# Author Correction: Targeting pleuro-alveolar junctions reverses lung fibrosis in mice

**DOI:** 10.1038/s41467-025-57261-3

**Published:** 2025-02-24

**Authors:** Adrian Fischer, Wei Han, Shaoping Hu, Martin Mück-Häusl, Juliane Wannemacher, Safwen Kadri, Yue Lin, Ruoxuan Dai, Simon Christ, Yiqun Su, Bikram Dasgupta, Aydan Sardogan, Christoph Deisenhofer, Subhasree Dutta, Amal Kadri, Tankut Gökhan Güney, Donovan Correa-Gallegos, Christoph H. Mayr, Rudolf Hatz, Mircea Gabriel Stoleriu, Michael Lindner, Anne Hilgendorff, Heiko Adler, Hans-Günther Machens, Herbert B. Schiller, Stefanie M. Hauck, Yuval Rinkevich

**Affiliations:** 1 Institute for Diabetes and Obesity (IDO), Helmholtz Diabetes Center (HDC), Helmholtz Zentrum München, Neuherberg, Germany; 2 Institute of Regenerative Biology and Medicine(IRBM), Helmholtz Zentrum München, Munich, Germany; 3 Member of the German Center of Lung Research (DZL), Munich, Germany; 4 Faculty of Medicine, Ludwig-Maximilians-University Munich, Munich, Germany; 5 Zhangzhou Health Vocational College, Zhangzhou, China; 6 Helmholtz Munich, Research Unit for Precision Regenerative Medicine (PRM), Member of the German Center for Lung Research (DZL), Munich, Germany; 7 Institute for Stroke and Dementia Research (ISD), LMU University Hospital, LMU Munich, Munich, Germany; 8 Asklepios Fachkliniken in Munich-Gauting, Munich, Germany; 9 University Department of Visceral and Thoracic Surgery Salzburg, Paracelsus Medical University, Salzburg, Austria; 10 Helmholtz Zentrum München, Institute of Lung Biology & Disease, Group Mechanism of Neonatal Chronic Lung Disease, Member of the German Center of Lung Research (DZL), Munich, Germany; 11 Comprehensive Pneumology Center with the CPC-M bioArchive and Institute of Lung Health and Immunity, Helmholtz-Zentrum München, Member of the German Center of Lung Research (DZL), Munich, Germany; 12 Institute of Asthma and Allergy Prevention, Helmholtz Zentrum München, German Research Center for Environmental Health, Neuherberg, Germany; 13 Walther-Straub-Institute of Pharmacology and Toxicology, Ludwig-Maximilians-University Munich, Munich, Germany; 14 Department of Plastic and Hand Surgery, Technical University of Munich, School of Medicine and Health, Klinikum rechts der Isar, Munich, Germany; 15 Institute of Experimental Pneumology, LMU University Hospital, Ludwig-Maximilians University, Munich, Germany; 16 Metabolomics and Proteomics Core, Helmholtz Zentrum München, Munich, Germany; 17 Institute of Regenerative Biology and Medicine, Chinese Institutes for Medical Research, Beijing, China; 18 Capital Medical University, Beijing, China

**Keywords:** Respiratory tract diseases, Alveolar macrophages

Correction to: *Nature Communications* 10.1038/s41467-024-55596-x, published online 02 January 2025

In this article the author’s name Martin Mück-Häusl was incorrectly written as Martin Mück Häusl. The original article has been corrected.

