# Supplementary material for: Targeting pleuro-alveolar junctions reverses lung fibrosis in mice
Source: Nat Commun. 2025 Jan 2;16:173. doi: 10.1038/s41467-024-55596-x (PMC11696612; doi:10.1038/s41467-024-55596-x)
Supplement: Supplementary file 1 — Supplementary Information [file 41467_2024_55596_MOESM1_ESM.pdf]

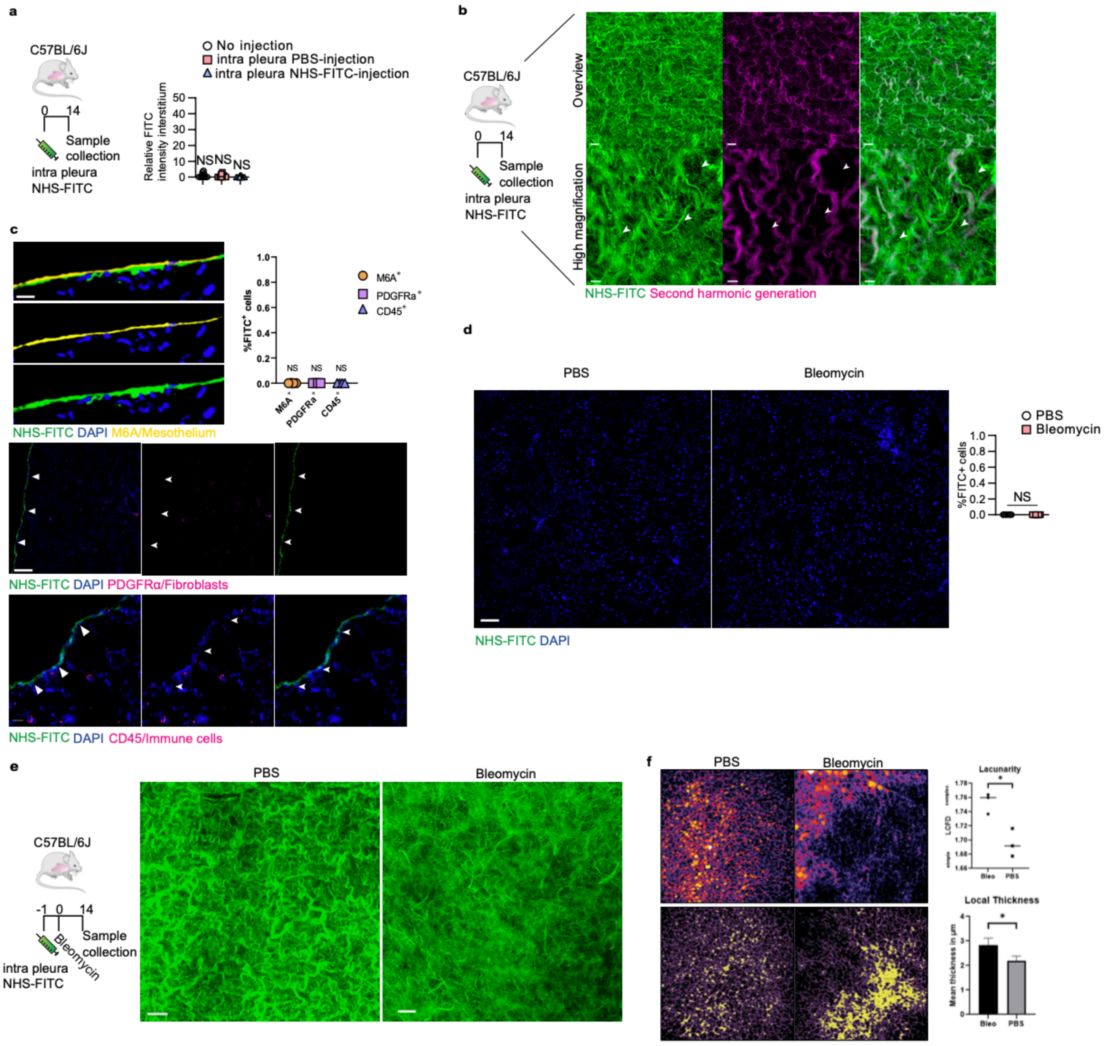

Supplementary Figure 1: Bleomycin induces structural changes in pleural surfaces.

a) Quantification of interstitial FITC Signal in mouse lungs 14 days post-intra-pleural injections. No injection acted as control. n = 10 biological replicates (C57BL/6J WT mice) and 10 independent experiments. Data represented are mean ± SD. One-way ANOVA was used for the multiple comparison (NS= not significant). b) Representative multiphoton images of NHS-FITC labelled murine lung surfaces. n = 6 biological replicates (C57BL/6J WT mice) and 6 independent experiments. Scale bars: Overview 15 µm; High magnification 5 µm. c) Representative immunolabeling histology images of NHS-FITC labelled mouse lungs PDGFRα acted as marker for fibroblasts, M6A for mesothelial cells and CD45 for immune cells. Fluorescence images of murine lungs, n = 6 biological replicates (C57BL/6J WT mice) and 6 independent experiments. Data represented are mean ± SD. Single comparison was performed by two-sided independent t-test. Scale bar: M6A 15 µm, PDGFRα 50 µm, CD45 20 µm. d) Representative histology images of mouse lungs treated with PBS and livers treated with bleomycin. e) Representative histology images of mouse lungs. Mice were intrapleurally injected with NHS-FITC labelling mix. The next day bleomycin or PBS control was installed. n = 6 biological replicates (C57BL/6J WT mice) and 6

independent experiments. Scale bars: 50 µm. f) Representative multiphoton images of murine lung surfaces 14 days p.b.i.. n = 3 biological replicates (C57BL/6J WT mice) and 3 independent experiments. Structural changes in connective tissues are visualized through changes in fractal dimensions, and calculated using Fiji/FracLac. Data represented are median ± SD. A two-sided independent t-test was used for the comparison of two groups(* $P<0.05$). Scale bars: 15 µm.

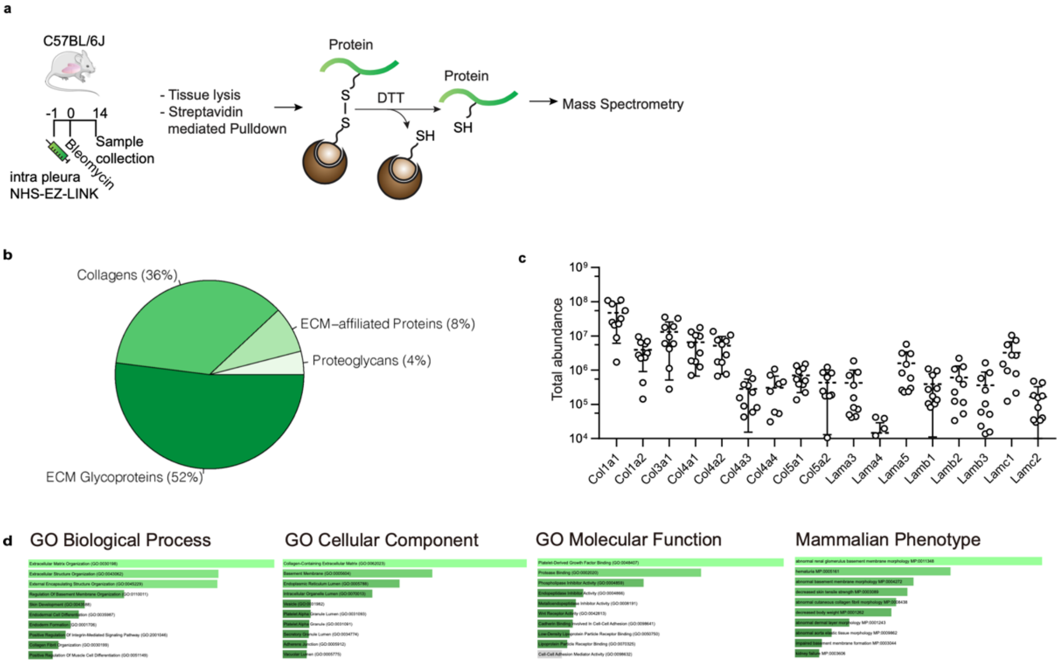

Supplementary Figure 2: Transferred matrix resembles scar tissue

a) Schematic representation of the mass spectrometry-based workflow to identify NHS-ester^{+} proteins. Schematic representation of pleural matrix fate mapping setup. C57BL/6J mice were intrapleurally injected with NHS-EZ-LINK labelling mix and two weeks later lungs were harvested. Lungs were lysed and proteins enriched by streptavidin-mediated pulldown. Proteins were eluted by changing the redox state of the EZ-LINK-Ester. Eluted proteins were analyzed by mass spectrometry. b) Distribution of matrisome proteins identified in mass spectrometry analysis. n = five biological replicates (C57BL/6J WT mice) and 5 independent experiments. c) Total abundances of collagens, fibrillar- and basement-elements. n = five biological replicates (C57BL/6J WT mice) and 5 independent experiments. Data represented are mean ± SD. d) Gene ontology analysis of matrisome genes via EnrichR webtool. Data represents the combined score. n = five biological replicates (C57BL/6J WT mice) and 5 independent experiments.

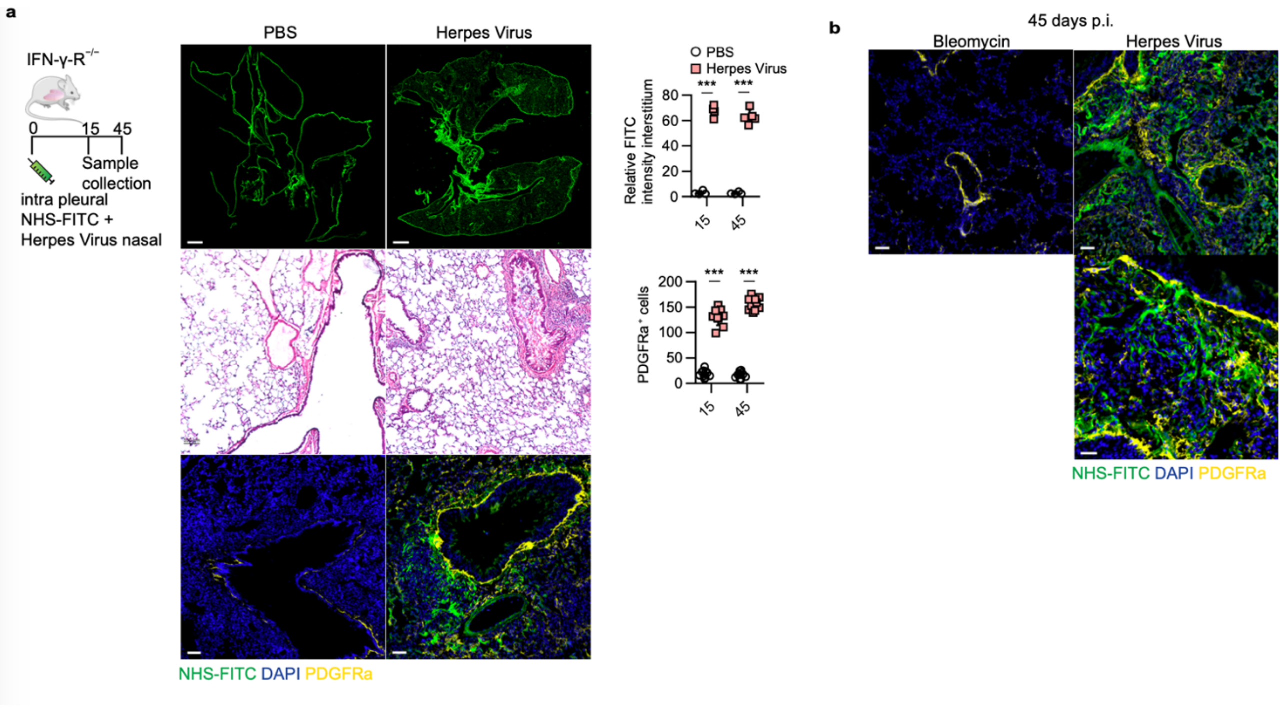

Supplementary Figure 3: Viral infection induces matrix flux

a) Representative histology images of mouse lungs 15 and 45 days post-herpes simples virus installation (p.hsv.i.). IFN-γ-R$^{-/-}$ mice were injected intrapleurally with NHS-FITC labelling mix; herpes virus was applied intra-nasally and mice were sacrificed on day 15 or 45. n = three biological replicates (IFN-γ-R$^{-/-}$ mice) and three independent experiments. Scale bars: 1000 µm; High magnification (100 µm). b) Representative histology images of mouse lungs 45 days post-bleomycin or herpes simples virus installation. n = 4 biological replicates and 4 independent experiments. Scale bars: 1000 µm; High magnification (50 µm). Data represented are mean ± SD. A two-sided independent t-test was used for the comparison of two groups (*** P<0.001).

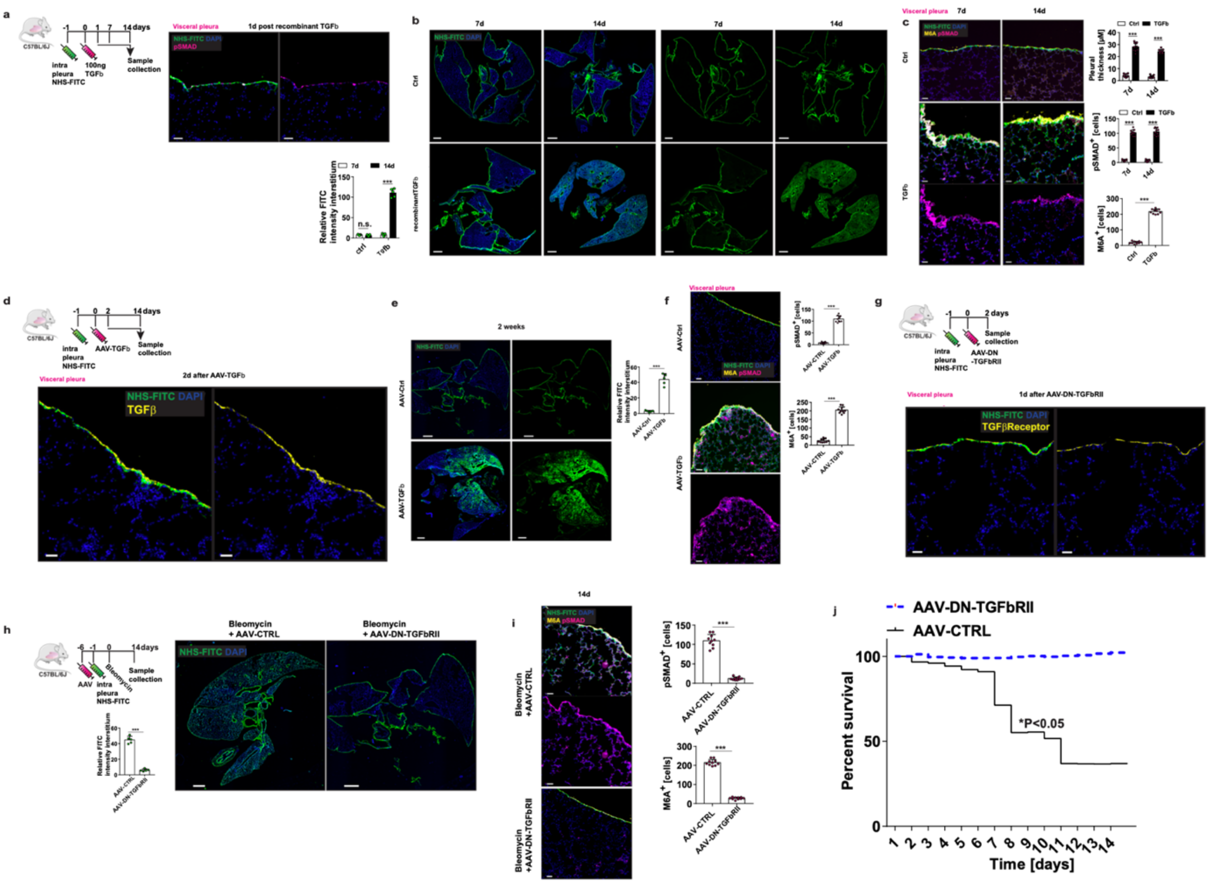

Supplementary figure 4: TGFβ Induced Lung Fibrosis Model and Effects

(a) Workflow of TGFβ induced lung fibrosis model. Mice were intrapleurally injected with NHS-FITC labelling mix. The next day 100ng of recombinant TGFβ was injected, leading to increased mesothelial TGFβ signaling. n = 5 biological replicates and 5 independent experiments. A two-sided independent t-test was used for the comparison of two groups (n.s. = not significant; *** P<0.001). (b) Active mesothelial TGFβ signaling leads to matrix invasion 14 days post recombinant TGFβ injection. n = 5 biological replicates and 5 independent experiments. Scale bars: 1000μM. (c) A single injection of recombinant TGFβ induced persistent active mesothelial TGFβ signaling and increased the number of M6A+ cells in lung interstitium. Scale bars: 50μM. A two-sided independent t-test was used for the comparison of two groups (*** P<0.001). (d) AAV based particles encoding for active TGFβ increase mesothelial TGFβ levels. n = 3 biological replicates and 3 independent experiments. Scale bars: 50μM. (e) Mesothelial TGFβ activation drives pulmonary fibrosis. AAV particles encoding for active TGF-β were applied intrapleurally. n = 5 biological replicates and 5 independent experiments. Scale bars: 1000μM. A two-sided independent t-test was used for the comparison of two groups (*** P<0.001). (f) Mesothelial TGFβ activation leads to increased interstitial pSMAD levels and M6A+ cells. n = 5 biological replicates and 5 independent experiments. Scale bars: 50μM. A two-sided independent t-test was used for the comparison of two groups (*** P<0.001). (g) AAV based particles encoding for dominant negative TGFβ receptors(AAV-DN-TGFbRII) express robustly in mesothelial cells. n = 3 biological replicates and 3 independent experiments.. Scale bars: 50μM. (h) Targeted inhibition of TGFβ in mesothelial cells blocks bleomycin-induced invasion

of fluid matrix. AAV particles encoding for dominant negative TGFβ receptors were applied intrapleurally; five days later NHS-FITC was installed intrapleurally; then one day later bleomycin was installed; 14 days after bleomycin installation organs were harvested. n = 5 biological replicates and 5 independent experiments. Scale bars: 1000μM. (i) Inhibition of mesothelial TGFβ blocked bleomycin-induced TGFβ signaling and increased the number of M6A+ cells in lung interstitium. n = 5 biological replicates and 5 independent experiments. Scale bars: 50μM. A two-sided independent t-test was used for the comparison of two groups (*** $P<0.001$). (j) Inhibition of mesothelial TGFβ prevents bleomycin-induced mortality. n = 5 biological replicates and 5 independent experiments. Log-rank test was used for statistical comparison. Data represented are mean ± SD.

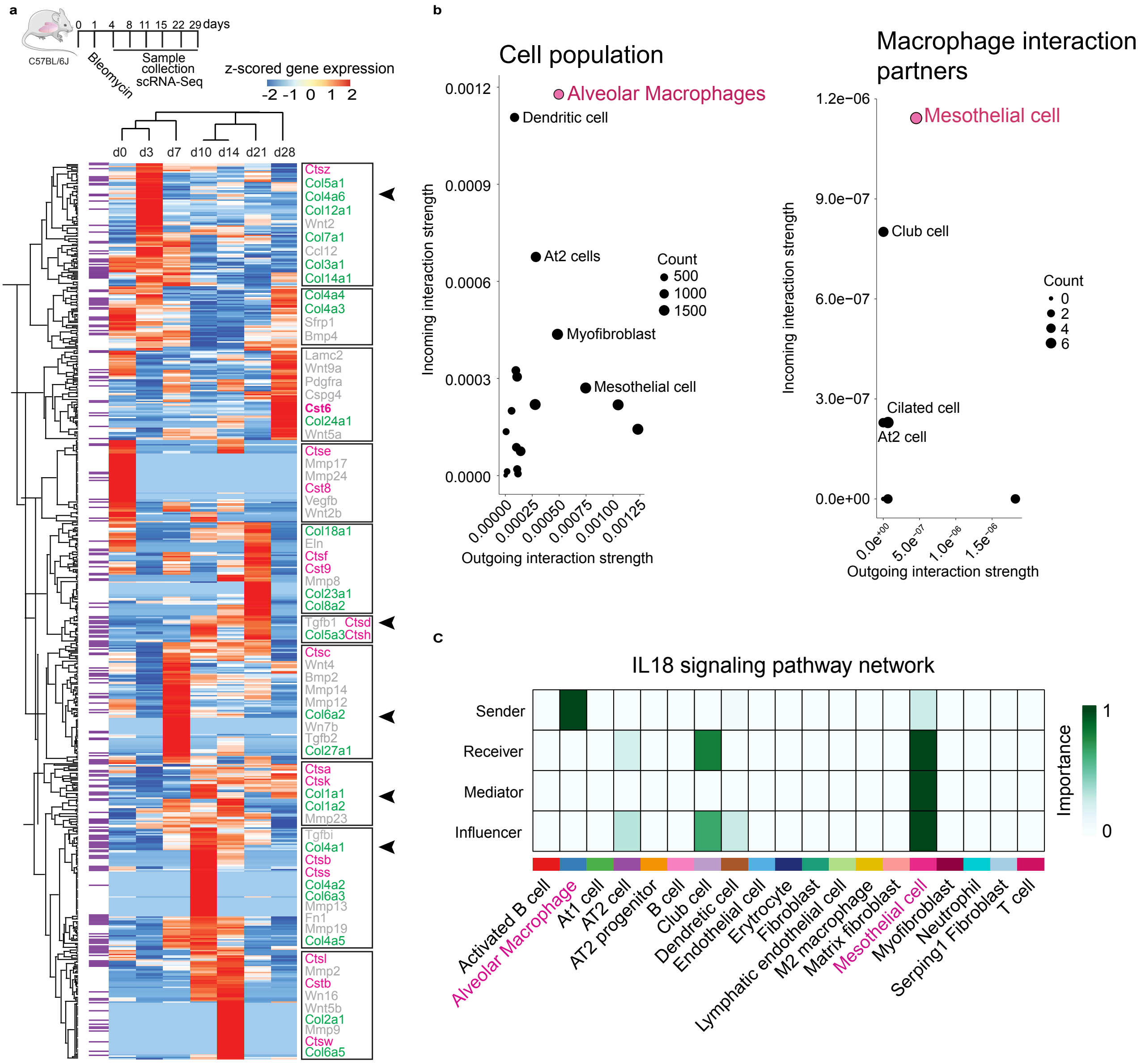

Supplementary Figure 5: Analysis of the scRNAseq datasets of murine lung mesothelium

a) Schematic and heatmap of scRNAseq datasets of murine lung mesothelium on days 0, 3, 7, 10, 14, 21 and 28 post-bleomycin installation. b) Graphic visualization of outgoing and incoming signals of identified cell populations in bleomycin treated animals (left) and alveolar macrophages (right). c) Heatmap visualization of the IL-18 signaling axis in bleomycin treated animals.

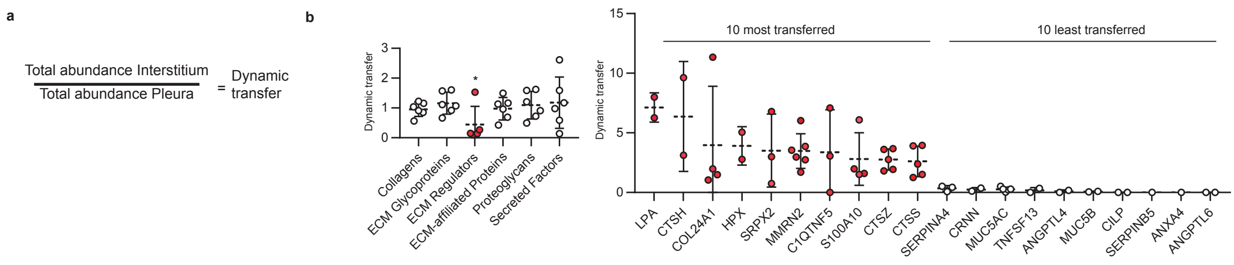

Supplementary Figure 6: Distinct transfer profiles in human pleural matrix

a) Calculation of translocation factor. Total abundance of proteins in the interstitium is divided by the amount detected in the pleural fraction of mass spectrometry analysis described in figure 4. b) Translocation factors of different proteins and matrisome families. n = five biological replicates and 5 independent experiments. Data represented are mean ± SD. Statistical comparison was performed using two-sided independent t-test (* P<0.05).

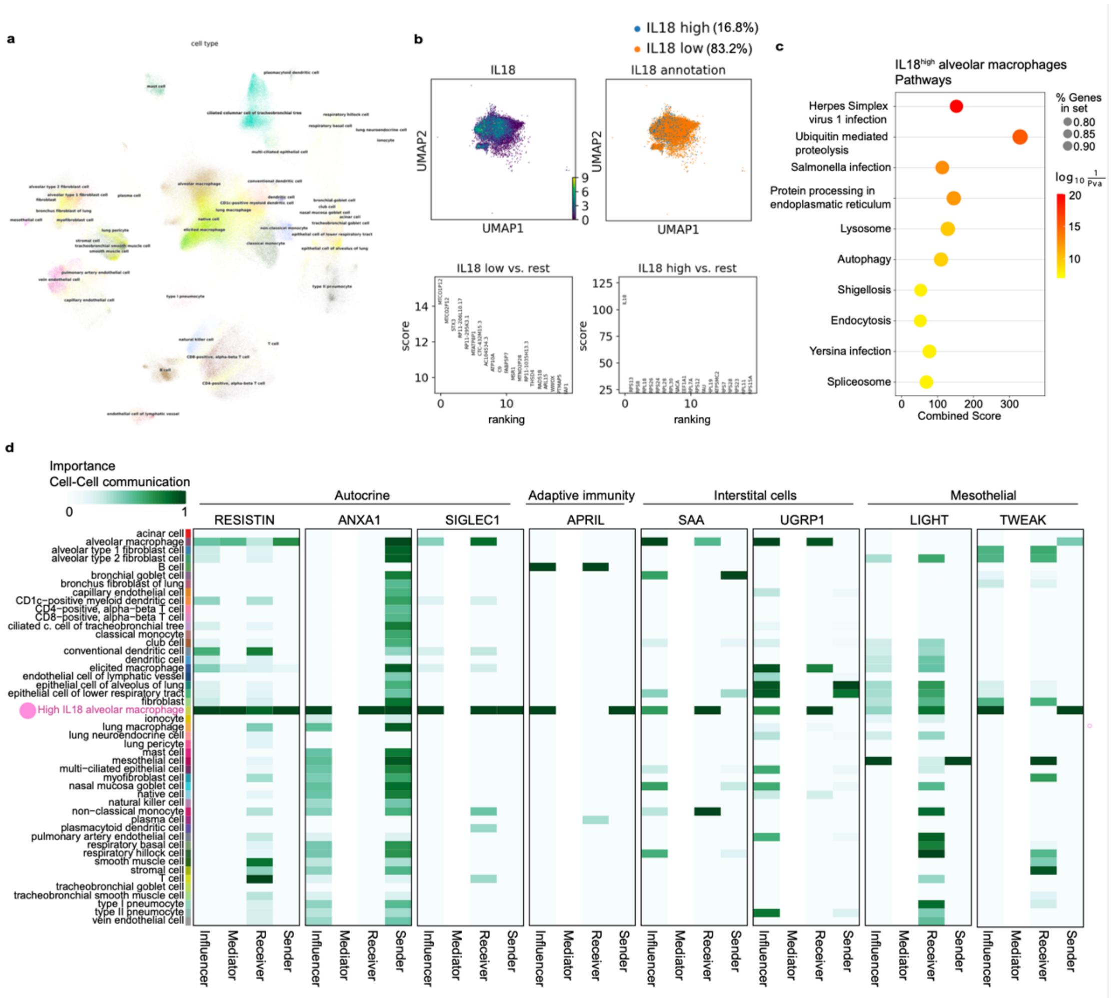

Supplementary Figure 7: Human IPF IL-18^{high} alveolar macrophages have distinct communication patterns and are in an increased infection-combating state

a) UMAPs of cell populations in human IPF samples. b) UMAP visualization of alveolar macrophages in human IPF with IL-18^{high} clustering and comparison of differentially expressed genes between all- and IL-18^{high} alveolar macrophages. c) Gene enrichment analysis of IL-18^{high} alveolar macrophages. d) Visualization of cell- to cell communication in human IPF, IL-18^{high} alveolar macrophages are highlighted in pink.

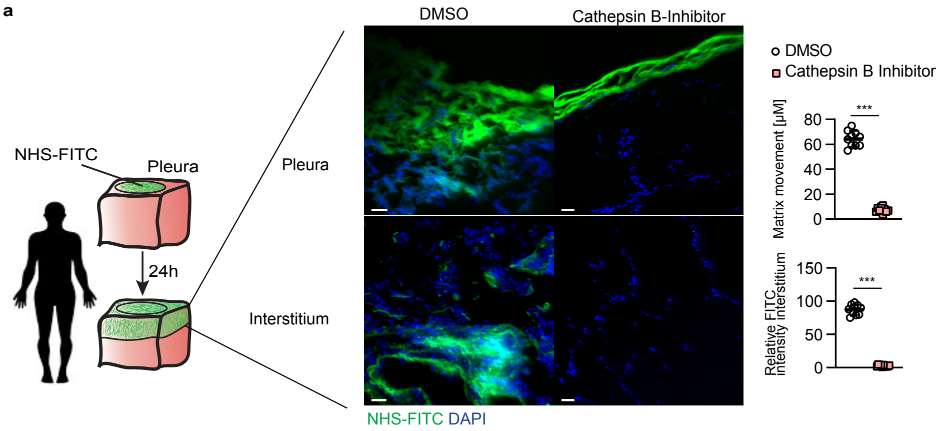

Supplementary Figure 8: Matrix transfer is blocked in human IPF lung samples

a) Representative histology images of human lung tissue with NHS-FITC+ marked pleural site cultivated ex vivo in presence of cathepsin b inhibitor or DMSO control respectively. Data represented are mean ± SD. n = six biological replicates and six independent experiments. Statistical comparison was performed by two-sided independent t-test (*** P<0.001). Scale bars: 50 µm.
