# Supplementary material for: Targeting pleuro-alveolar junctions reverses lung fibrosis in mice
Source: Nat Commun. 2025 Jan 2;16:173. doi: 10.1038/s41467-024-55596-x (PMC11696612; doi:10.1038/s41467-024-55596-x)
Supplement: Supplementary file 2 — Description of Additional Supplementary Files [file 41467_2024_55596_MOESM2_ESM.docx]

Description of Additional Supplementary Files

File Name: Supplementary Data 1

Description: Mice mass spec data

File Name: Supplementary Data 2

Description: Human mass spec data

File Name: Supplementary Video 1

Description: Intra-pleural injection of NHS-FITC labels whole lung surface. Green: NHS-FITC; Red: anti-CD45; magenta anti-αSMA.

File Name: Supplementary Video 2

Description: High-magnification 3D imaging of lung surfaces reveals rigid collagenous frames (magenta) and volumes of protein-rich immature matrix (green) on murine lung surfaces. Green: NHS-FITC; Magenta: Second harmonics generation.

File Name: Supplementary Video 3

Description: Bleomycin-induced pneumonia leads in turn to invasion of pleural matrix elements. Green: NHS-FITC; Red: anti-CD45; magenta anti-αSMA.

File Name: Supplementary Video 4

Description: High-magnification 3D imaging of lung surfaces after bleomycin injection. Green: NHS-FITC; Magenta: Second harmonics generation.

File Name: Supplementary Video 5

Description: High-magnification 3D imaging of human diseased lung. Streams of protein-rich matrix invading the lungs. Green: NHS-FITC; Magenta: Second harmonics generation.

File Name: Supplementary Video 6

Description: High-magnification 3D imaging of human diseased lung. Streams of protein-rich matrix invading bronchial compartments. Green: NHS-FITC; Magenta: Second harmonics generation.
