# Supplementary material for: Targeting pleuro-alveolar junctions reverses lung fibrosis in mice
Source: Nat Commun. 2025 Jan 2;16:173. doi: 10.1038/s41467-024-55596-x (PMC11696612; doi:10.1038/s41467-024-55596-x)
Supplement: Supplementary file 11 — Reporting Summary [file 41467_2024_55596_MOESM11_ESM.pdf]

Corresponding author(s): Yuval Rinkevich, Wei Han

Last updated by author(s): Jul 29, 2024

Reporting Summary

Nature Portfolio wishes to improve the reproducibility of the work that we publish. This form provides structure for consistency and transparency in reporting. For further information on Nature Portfolio policies, see our Editorial Policies and the Editorial Policy Checklist.

Statistics

For all statistical analyses, confirm that the following items are present in the figure legend, table legend, main text, or Methods section.

n/a	Confirmed	
☐	☒	The exact sample size (*n*) for each experimental group/condition, given as a discrete number and unit of measurement
☐	☒	A statement on whether measurements were taken from distinct samples or whether the same sample was measured repeatedly
☐	☒	The statistical test(s) used AND whether they are one- or two-sided<br>*Only common tests should be described solely by name; describe more complex techniques in the Methods section.*
☒	☐	A description of all covariates tested
☒	☐	A description of any assumptions or corrections, such as tests of normality and adjustment for multiple comparisons
☐	☒	A full description of the statistical parameters including central tendency (e.g. means) or other basic estimates (e.g. regression coefficient) AND variation (e.g. standard deviation) or associated estimates of uncertainty (e.g. confidence intervals)
☐	☒	For null hypothesis testing, the test statistic (e.g. *F*, *t*, *r*) with confidence intervals, effect sizes, degrees of freedom and *P* value noted<br>*Give P values as exact values whenever suitable.*
☒	☐	For Bayesian analysis, information on the choice of priors and Markov chain Monte Carlo settings
☒	☐	For hierarchical and complex designs, identification of the appropriate level for tests and full reporting of outcomes
☒	☐	Estimates of effect sizes (e.g. Cohen's *d*, Pearson's *r*), indicating how they were calculated

Our web collection on statistics for biologists contains articles on many of the points above.

Software and code

Policy information about availability of computer code

Data collection	Axiovision v4.8.2; Leica Application Suite v4.8
Data analysis	GraphPad v.7.0 ; Imaris v9.1.3.; Microsoft365 ; Zen v2.1; Enrichr webtool; Mascot Daemon 2.6.2. ; Progenesis Qisoftware v4.1 ; Axiovision v4.8.2; Leica Application Suite v4.8

For manuscripts utilizing custom algorithms or software that are central to the research but not yet described in published literature, software must be made available to editors and reviewers. We strongly encourage code deposition in a community repository (e.g. GitHub). See the Nature Portfolio guidelines for submitting code & software for further information.

Data

Policy information about availability of data

All manuscripts must include a data availability statement. This statement should provide the following information, where applicable:

- Accession codes, unique identifiers, or web links for publicly available datasets
- A description of any restrictions on data availability
- For clinical datasets or third party data, please ensure that the statement adheres to our policy

Computer codes are available from the corresponding author on request.

Research involving human participants, their data, or biological material

Policy information about studies with human participants or human data. See also policy information about sex, gender (identity/presentation), and sexual orientation and race, ethnicity and racism.

Reporting on sex and gender	sex and gender were not considered in the study design; sex and gender of participants was determined based on self-report.
Reporting on race, ethnicity, or other socially relevant groupings	race and ethnicity were not considered in the study design; race and ethnicity of participants was determined based on self-report.
Population characteristics	Donors underwent lung surgeries with diagnosis of lung cancer or IPF.
Recruitment	Human samples were collected from the hospital, including peritumor and IPF samples.
Ethics oversight	All experimental procedures were performed in accordance with the Ethics committee vote number / Parent Proposal number: BA34/2018.

Note that full information on the approval of the study protocol must also be provided in the manuscript.

Field-specific reporting

Please select the one below that is the best fit for your research. If you are not sure, read the appropriate sections before making your selection.

[x] Life sciences [ ] Behavioural & social sciences [ ] Ecological, evolutionary & environmental sciences

For a reference copy of the document with all sections, see nature.com/documents/nr-reporting-summary-flat.pdf

Life sciences study design

All studies must disclose on these points even when the disclosure is negative.

Sample size	No statistical method was used to predetermine sample size. Required experimental sample sizes were estimated based on previous established protocols in the field. The sample sizes were adequate as the differences between experimental groups were reproducible. All n values are clearly indicated within the figure legends.
Data exclusions	No data was excluded from the analysis
Replication	All experiments were performed at least three times with similar results.
Randomization	Mice were randomly divided into treatment groups.
Blinding	No experiments presented in this study required blinding.

Reporting for specific materials, systems and methods

We require information from authors about some types of materials, experimental systems and methods used in many studies. Here, indicate whether each material, system or method listed is relevant to your study. If you are not sure if a list item applies to your research, read the appropriate section before selecting a response.

Materials & experimental systems

n/a	Involved in the study	
[ ]	[x]	Antibodies
[x]	[ ]	Eukaryotic cell lines
[x]	[ ]	Palaeontology and archaeology
[ ]	[x]	Animals and other organisms
[x]	[ ]	Clinical data
[x]	[ ]	Dual use research of concern
[x]	[ ]	Plants

Methods

n/a	Involved in the study	
[x]	[ ]	ChIP-seq
[x]	[ ]	Flow cytometry
[x]	[ ]	MRI-based neuroimaging

Antibodies

Antibodies used	Primary antibody<br>Anti-PDGFRa(R&D systems; AF1062;1:100);<br>Anti-YAP/TAZ(Abcam; ab205270;1:100);

Anti-αSMA(Abcam; ab5694;1:100);
Anti-CD45(Abcam; ab10558;1:100);
Anti-pSMAD(Cell Signaling Technology;18338S;1:100);
Anti-M6A(Biozol Diagnostica; MBL-D055-3;1:150);
Anti CD11c(Abcam; ab254183;1:200);
Anti-IL18R(Invitrogen; A21081;1:200);
Anti-IL18(Genetex; GTX637411;1:200);
Anti-pan Collagen(LIFE TECHNOLOGIES;PA185324;1:200);
Anti-Cathepsin B (Elabscience; E-AB-10208.60;1:100).

Secondary antibody
AlexaFluor568-, AlexaFluor594-, or AlexaFluor647-conjugated secondary antibodies against suitable species (Life technologies, 1:500)

Validation	See manufacturers' notes. Antibodies were additionally validated using respective isotype antibodies in immunofluorescence assays.

Animals and other research organisms

Policy information about studies involving animals; ARRIVE guidelines recommended for reporting animal research, and Sex and Gender in Research

Laboratory animals	C57BL/6J WT mice ; IFN-γ-R−/− mice .
Wild animals	The study did not involve wild animals.
Reporting on sex	sex was not considered in the study design.
Field-collected samples	The study did not involve samples collected from field.
Ethics oversight	Government of Upper Bavaria , Germany.

Note that full information on the approval of the study protocol must also be provided in the manuscript.

Plants

Seed stocks	*Report on the source of all seed stocks or other plant material used. If applicable, state the seed stock centre and catalogue number. If plant specimens were collected from the field, describe the collection location, date and sampling procedures.*
Novel plant genotypes	*Describe the methods by which all novel plant genotypes were produced. This includes those generated by transgenic approaches, gene editing, chemical/radiation-based mutagenesis and hybridization. For transgenic lines, describe the transformation method, the number of independent lines analyzed and the generation upon which experiments were performed. For gene-edited lines, describe the editor used, the endogenous sequence targeted for editing, the targeting guide RNA sequence (if applicable) and how the editor was applied.*
Authentication	*Describe any authentication procedures for each seed stock used or novel genotype generated. Describe any experiments used to assess the effect of a mutation and, where applicable, how potential secondary effects (e.g. second site T-DNA insertions, mosiacism, off-target gene editing) were examined.*
